# The impact of pulmonary hypertension on prognosis in moderate-to-severe mitral regurgitation patients treated with transcatheter edge-to-edge mitral valve repair: a comprehensive meta-analysis

**DOI:** 10.3389/fcvm.2024.1489674

**Published:** 2025-01-10

**Authors:** Zhili Wei, Xinquan Shao, Zhijing An, Yixuan Chang, Shidong Liu, Ziran Luo, Yang Chen, Bing Song

**Affiliations:** ^1^ The First Clinical Medical College of Lanzhou University, Lanzhou University, Lanzhou, China; ^2^ Department of Cardiovascular Surgery, First Hospital of Lanzhou University, Lanzhou, China

**Keywords:** transcatheter edge to edge mitral valve repair, mitral valve regurgitate, pulmonary hypertension, meta-analysis, prognosis

## Abstract

**Objective:**

This meta-analysis aims to assess the impact of pulmonary hypertension (PH) on the clinical prognosis of patients with moderate to severe mitral valve regurgitation (MR) undergoing transcatheter edge-to-edge mitral valve repair (TEER).

**Methods:**

As of August 2024, relevant studies were identified through searches of the PubMed, Cochrane Library, Web of Science, and Embase databases. A comprehensive screening process was conducted, with quality assessment performed utilizing the Newcastle Ottawa Scale (NOS). Data analysis was carried out using Stata17.0 software, generating forest plots, funnel plots, Egger's test, and sensitivity analysis plots to analyze heterogeneity and publication bias. Odds ratios (OR) and standardized mean differences (SMD) were calculated for dichotomous and continuous variables, respectively, each presented with a 95% confidence interval (CI).

**Results:**

A total of 10 studies involving 28,404 patients were included, with all articles achieving a NOS score of ≥7. The outcome indicators were as follows: 2-year all-cause mortality [OR = 2.06, 95%CI(1.49, 2.84), *p* < 0.01, *I*^2^ = 79.9%]; heart failure rehospitalization rate [OR = 1.56, 95%CI(1.29,1.76), *p* < 0.01, *I*^2^ = 41.7%]; 30-day all-cause mortality [OR = 2.10, 95%CI(1.78,2.47), *p* < 0.01, *I*^2^ = 0%]; cardiogenic mortality [OR = 2.00, 95%CI (1.61,2.49), *p* < 0.01, *I*^2^ = 0%]; and length of hospital stay [OR = 0.17, 95%CI(0.14,0.20), *p* < 0.01, *I*^2^ = 0%]. All outcome indicators demonstrated that the PH group had significantly worse outcomes compared to the non-PH group. Subgroup analyses were performed on outcome indicators with notable heterogeneity, focusing on PH measurement methods, PH diagnostic criteria, and the severity of PH. The results indicated that most combined subgroup outcomes were consistent with the overall findings and showed significantly reduced heterogeneity. The sources of heterogeneity are likely attributed to the methods of PH measurement, diagnostic criteria for PH, and the severity of PH.

**Conclusion:**

Within two years after undergoing transcatheter edge-to-edge repair (TEER), patients with MR and PH experiecne significantly higher rates of all-cause mortality, 30-day all-cause mortality, heart failure readmissions, cardiogenic mortality, and longer hospital stays compared to those without PH.

**Systematic Review Registration:**

https://inplasy.com/, identifier (INPLASY202480068).

## 1 Introduction

Mitral valve regurgitation (MR) occurs when the heart contracts, causing the mitral valve to fail to close completely, allowing blood to flow back from the left ventricle into the left atrium. It is one of the most common and severe valvular heart diseases in the cardiovascular field (1). In Western countries, over 2 million Americans are affected by mitral regurgitation (2). Surgical cases of MR often result from degenerative diseases, coronary artery disease, rheumatic disease, or endocarditis. Degenerative diseases are the most common cause, accounting for 60%–70% of cases, usually associated with mitral valve prolapse, though isolated mitral annular calcification can also occur (3, 4). MR can also be caused by inflammatory diseases, cardiomyopathies, trauma, congenital factors, or medications. Ischemic MR, typically caused by coronary artery disease, accounts for 20% of cases (5). Endocarditis and rheumatic disease each contribute to 2%–5% of MR cases (6–8). The prevalence of MR increases with age, and with the aging population, the number of affected patients is projected to rise by 2030 (2). MR can be categorized based on pathophysiological mechanisms into functional and organic MR. Functional MR involves valvular deformation due to ventricular remodeling while the valve structure itself remains normal. In contrast, organic MR involves intrinsic valvular disease (5). MR increases the volume load on the left atrium and ventricle, which can lead to myocardial dilative cardiomyopathy and dysfunction of the left atrium and ventricle over time, potentially causing heart failure (9, 10). Open heart surgery for valve repair or replacement is the primary treatment for severe mitral regurgitation. However, for elderly patients and those at high risk, open surgery carries significantly higher complications and mortality rates. The MitraClip (Abbott Vascular, CA, USA) for Transcatheter Edge-to-Edge Repair (TEER) is a new, safe, and minimally invasive method to treat MR, offering an alternative for patients who cannot tolerate traditional surgical. This approach has demonstrated effectiveness in reducing MR severity and improving symptoms (11–13).

Pulmonary arterial hypertension (PH) is characterized by an abnormal increase in pulmonary artery pressure and can be classified into several types based on etiology: primary PH, PH secondary to pulmonary disease or hypoxemia, PH due to heart disease, chronic thromboembolic PH, and PH with unclear or multifactorial mechanisms (14). Approximately 1% of the global population is affected by PH, with a higher prevalence in low and middle-income countries (15). In patients with MR, PH is a common comorbidity, affecting about 23% of those with idiopathic moderate MR and up to 64% of those with severe MR (16). Without timely treatment, sustained high pulmonary artery pressure can lead to vasoconstriction and irreversible remodeling of the pulmonary arterioles (17–19). PH not only contributes to the occurrence and development of mitral regurgitation but also significantly influences treatment choices and prognosis. While transcatheter edge-to-edge repair (TEER) has shown promise in MR treatment, its effectiveness may be compromised in patients with MR with concurrent PH. Research indicates that PH exacerbates the hemodynamic burden on the heart, further deteriorating left and right ventricular function, which can reduce the success rate of TEER and increase the risk of surgical complications (20, 21). Elevated pulmonary artery pressures have also been linked to higher long-term mortality (17). Therefore, understanding the impact of PH on TEER effectiveness is crucial for improving the prognosis of MR patients.

## 2 Methods

We conducted systematic reviews following the guidelines of the Preferred Reporting Items for Systematic Reviews and Meta-Analyses (PRISMA) (22). The complete research protocol was officially registered in the INPLASY database (International Platform of Registered Systematic Review and Meta-analysis Protocols) under registration number INPLASY202480068. This study can be accessed at https://inplasy.com/, last accessed on August 15, 2024.

### 2.1 Search strategy

We searched PubMed, Cochrane Library, Web of Science, and Embase databases for peer-reviewed articles and clinical studies on the impact of PH on the surgical outcomes and prognosis of patients with moderate to severe mitral MR undergoing TEER. The search covered the period from the inception of the databases through August 2024 and was supplemented by manual searches for additional relevant articles. The search strategy utilized a combination of controlled vocabulary and free-text terms, including: edge-to-edge transcatheter mitral valve repair, mitral valve transcatheter edge-to-edge repair, TEER, Mitral Valve Insufficiency, mitral valve regurgitation, pulmonary hypertension, and Hypertension Pulmonary.

### 2.2 Study selection

The study selection process involved the following steps: Two researchers independently screened the publications and extracted data, then cross-checked each other's work for accuracy. Disagreements regarding the inclusion of specific studies were resolved by consulting a third party. The inclusion criteria were: (1) Cohort studies or randomized controlled trials (RCTs); (2) Patients diagnosed with moderate to severe MR by cardiac color Doppler ultrasound and undergoing TEER treatment; (3) Patients with MR diagnosed as having or not having PH using echocardiography or right heart catheterization; (4) Outcome indicators include: 2-year all-cause mortality, heart failure rehospitalization rate, 30-day all-cause mortality, cardiogenic mortality, and length of hospital stay. Exclusion criteria included: (1) Duplicate publications; (2) Conference abstracts or review articles; (3) inaccessible original texts, incomplete data, or results not relevant to the research question; (4) Case reports, letters, or review articles; (5) Non-clinical studies, such as cell experiments, meta-analyses, or animal studies.

### 2.3 Study endpoints

The study endpoints are categorized into primary and secondary endpoints. The primary endpoint is the 2-year all-cause mortality rate. Secondary endpoints include the heart failure rehospitalization rate, 30-day all-cause mortality rate, cardiogenic mortality rate, and length of hospital stay.

### 2.4 Data extraction

Data were extracted using standardized tables, including the following categories: (1) Basic information on the included studies and baseline data of the subjects: first author and year, sample size, type of study, age, gender, PH diagnostic criteria, and PH diagnostic methods. (2) Medical history, including condition such as diabetes, hypertension, coronary artery disease, chronic obstructive pulmonary disease (COPD), atrial fibrillation, and myocardial infarction, as well as procedures such as coronary artery bypass graft (CABG) and percutaneous coronary intervention. (3) Outcome indicators, including rates and measures such as 2-year all-cause mortality, heart failure rehospitalization rate, 30-day all-cause mortality, cardiogenic mortality, and length of hospital stay. (4) Information related to the quality assessment of the included literature and GRADE (Grading of Recommendations, Assessment, Development, and Evaluations), a system used to evaluate the quality of research.

### 2.5 Quality assessment

The studies included in this article are cohort studies, and thus the Newcastle Ottawa Scale (NOS) was used for quality assessment. This scale evaluates three primary dimensions: the selection of research subjects, comparability between groups, and outcome assessment. Studies scoring 7 points or higher are classified as high-quality, those with scores between 4 and 6 are considered medium-quality, and studies scoring less than 4 as low-quality. Additionally, two researchers independently conducted the quality assessments and cross-checked their evaluations. Any discrepancies were resolved through consensus or independent arbitration.

### 2.6 Statistical analysis

This study uses Stata17.0 software (Stata Corp LLC, College Station, TX, USA) to perform the meta-analysis, generating forest plots, sensitivity analysis plots, funnel plots, and conducting Egger's test. Odds ratio (*OR*) and Standardized Mean Difference (*SMD*) are used as the statistical measures for dichotomous and continuous variables, respectively, with each providing a 95% Confidence Interval (95%CI). The heterogeneity of the studies is assessed using *I*^2^ and *p*-values. Significant heterogeneity, indicated by *p* < 0.05 or *I*^2^ > 50%, necessitates the use of a random-effects model for the meta-analysis; otherwise, a fixed-effects model is applied. Upon significant heterogeneity is detected, its sources are explored through sensitivity and subgroup analyses. Funnel plots are used for qualitative assessment of publication bias, while Egger's test is employed for quantitative assessment.

### 2.7 GRADE evidence level assessment

The GRADE evidence quality level is assessed based on five key aspects: imprecision, risk of bias, indirectness, publication bias, and inconsistency. The results are classified into four levels: high, moderate, low, and very low, as detailed in the accompanying table.

## 3 Results

### 3.1 Study selection

**Figure 1:**
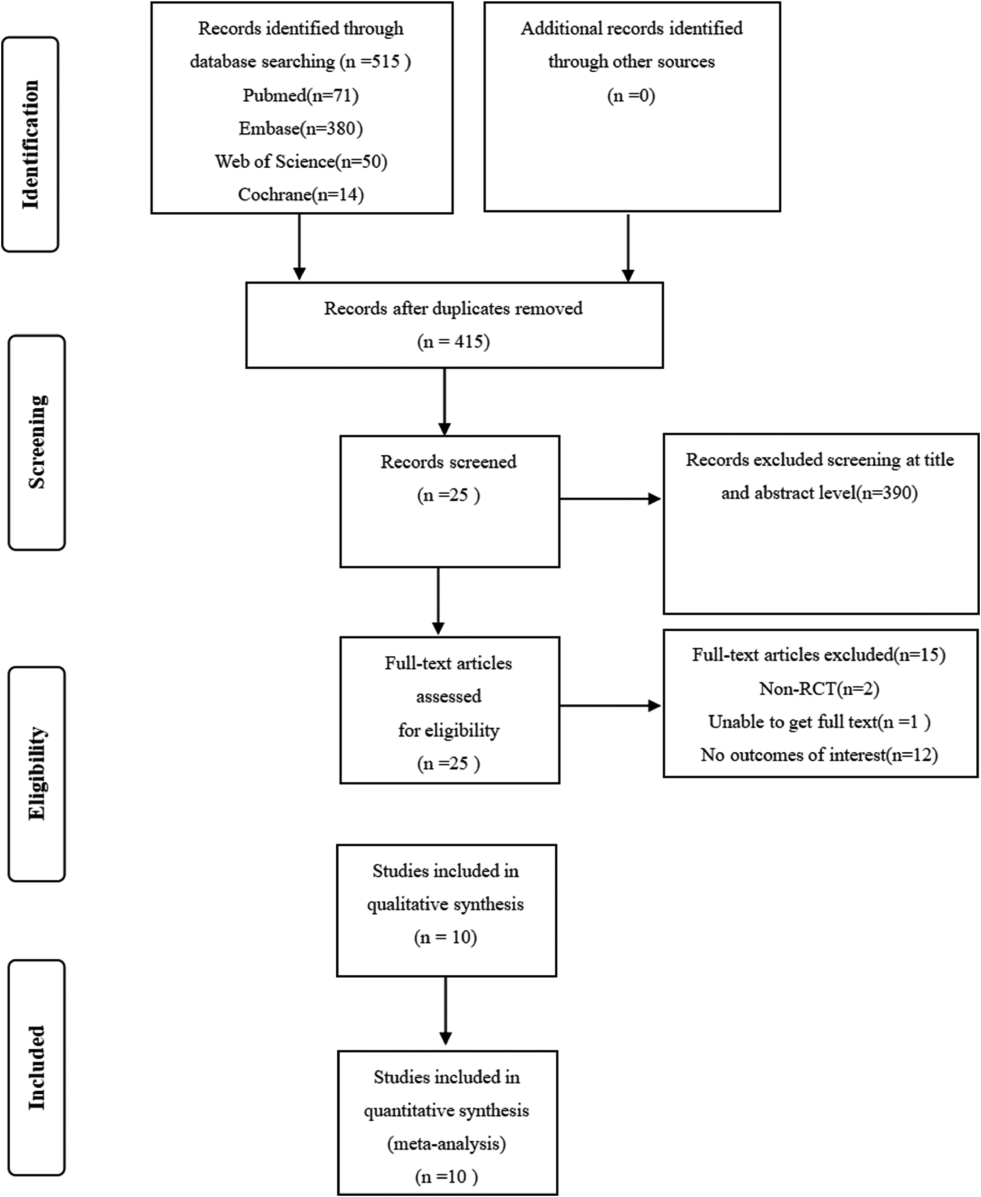
Flow chart of document retrieval.

From the PubMed, Cochrane Library, Web of Science, and Embase databases, a total of 515 articles were retrieved and imported into Endnote 21.0. After removing duplicates, 100 articles remained for further screening. Titles and abstracts were reviewed, leading to the preliminarily exclusion of 390 articles. A detailed review of the full texts was conducted for the remaining 25 articles. Among these, 2 were non-cohort studies (i.e., studies not following a group over time), 1 could not be accessed, and 12 did not provide results pertinent to our research focus. Ultimately, 10 articles (23–32) were included. Figure 1 illustrates the flowchart of the literature screening process.

### 3.2 Study characteristics and quality assessment

This study analyzed 10 articles involving 28,404 patients who underwent transcatheter edge-to-edge repair (TEER) for MR (23–32). All studies included were cohort studies. The risk of bias was assessed using the Newcastle-Ottawa Scale (NOS), with all articles scoring ≥7 on the NOS, indicating medium to high study quality. Details of the bias risk assessments for the studies are provided in the designated tables (Table 1).

**Table 1:** Newcastle-Ottawa scale.

Study	Selection	Comparability	Outcome	Total score
Matsumoto et al. (23)	★★★★	★★	★★★	9★
Khan et al. (24)	★★★	★★	★★★	8★
Ben-Yehuda et al. (25)	★★★★	★★	★★	8★
Rashi et al. (26)	★★★★	★	★★	7★
Tigges et al. (27)	★★★	★★	★★★	8★
Chaaya et al. (28)	★★★★	★	★★★	8★
Jaeger et al. (29)	★★★★	★★	★★★	9★
Ubben et al. 2024 (30)	★★★★	★	★★	7★
Ahmed et al. (31)	★★★	★★	★★★	8★
Al-Bawardy et al. (32)	★★★★	★★	★★★	9★

### 3.3 Patient characteristics

Specific baseline data for the patients included in the study are shown in Table 2. A total of 28,404 patients who received TEER for MR were analyzed. The average age of the patients was 76.0 years, with a range from 71.7 to 82.0 years. Females constituted 41.1% of the patient cohort, with a range from 28.6% to 49.4%. The following conditions were observed among the patients: 60.9% had coronary artery disease (range 31.4%–80.4%), 72.8% had hypertension (range 12.0%–89.6%), 31.1% had diabetes (range 10.0%–58.7%), 21.6% had chronic obstructive pulmonary disease (range 9.0%–45.8%), 55.9% had atrial fibrillation (AF) (range 39.0%–73.4%), 33.9% had myocardial infarction (range 21.3%–54.8%), 33.3% had a history of percutaneous coronary intervention (PCI) (range 8.6%–52.9%), and 29.7% had a history of coronary artery bypass graft (CABG) surgery (range 18.9%–45.8%). Patients were categorized into groups with PH and without pulmonary hypertension (Non-PH).

**Table 2:** Baseline features of patients.

Study	General characteristics					PH related data		Previous complications (%)						Previous surgical history (%)	
	Nation	Study design	Sample size (example)	Female (%)	Age (years)	PH test method	PH diagnostic criteria (mmHg)	CAD	Hypertension	Diabetes mellitus	COPD	AF	MI	PCI	CABG
Matsumoto et al. (23)	America	Cohort study	91	35.4/41.9	76.5/73.7	TTE	50	62.5/62.8	89.6/69.8	35.4/30.2	16.7/20.9	60.4/39.5	31.2/32.6	35.4/25.6	45.8/41.9
Khan et al. (24)	America	Cohort study	21,505	49.4/45.8	81.0/80.0	NR	NR	62.5/61.5	83.2/80.5	10.0/10.2	45.8/25.3	68.2/57.9	NR	NR	NR
Ben-Yehuda et al. (25)	America	Cohort study	528	30.4/40.4	72.9/71.7	TTE	50	NR	80.4/79.9	42.9/33.1	22.3/24.7	56.0/53.8	53.8/48.8	45.1/46.5	43.5/38.7
Rashi et al. (26)	Israel	Cohort study	177	40.0/54.6	74.5/73.8	TTE	35/36	66.5/45.5	87.7/82.0	41.9/23.0	14.8/9.0	48.4/39.0	54.8/39.0	52.9/33.0	26.5/27.3
Tigges et al. (27)	Germany	Cohort study	643	40.6/32.5	75.9/74.3	TTE	35/36	74.7/80.4	79.9/76.1	33.2/29.8	24.4/22.3	45.6/41.7	26.0/27.6	NR	22.6/29.6
Chaaya et al. (28)	America	Cohort study	114	38.0/28.6	74.6/75.0	RHC	NR	40.5/31.4	72.2/77.1	38.0/14.3	NR	73.4/48.6	22.8/22.9	27.9/8.6	22.8/25.7
Jaeger et al. (29)	Germany	Cohort study	238	36.2/43.4	77.3/75.2	RHC	24/25	76.8/66.0	80.5/77.4	30.8/26.4	11.4/9.4	69.7/71.7	NR	NR	19.5/18.9
Ubben et al. (30)	Germany	Cohort study	449	NR	NR	TTE	35/36	NR	NR	NR	NR	NR	NR	NR	NR
Ahmed et al. (31)	America	Cohort study	1037	48.4/41.5	75.1/74.1	NR	NR	NR	12.7/12.0	58.7/50.8	31.0/24.2	NR	NR	NR	NR
Al-Bawardy et al. (32)	America	Cohort study	4071	46.0/47.1	80.6/82.0	RHC	24/25	NR	86.1/83.2	29.1/21.6	NR	63.7/57.5	25.9/21.3	31.4/26.1	28.6/24.0

TTE, transthoracic echocardiography; RHC, right heart floating catheter; PH, pulmonary hypertension; COPD, chronic obstructive pulmonary disease; CABG, coronary artery bypass graft; PCI, percutaneous coronary intervention; CAD, coronary artery disease; AF, atrial fibrillation; MI, myocardial infarction; NR, not reported.

## 4 Outcomes

### 4.1 Two-years all-cause mortality

**Figure 2:**
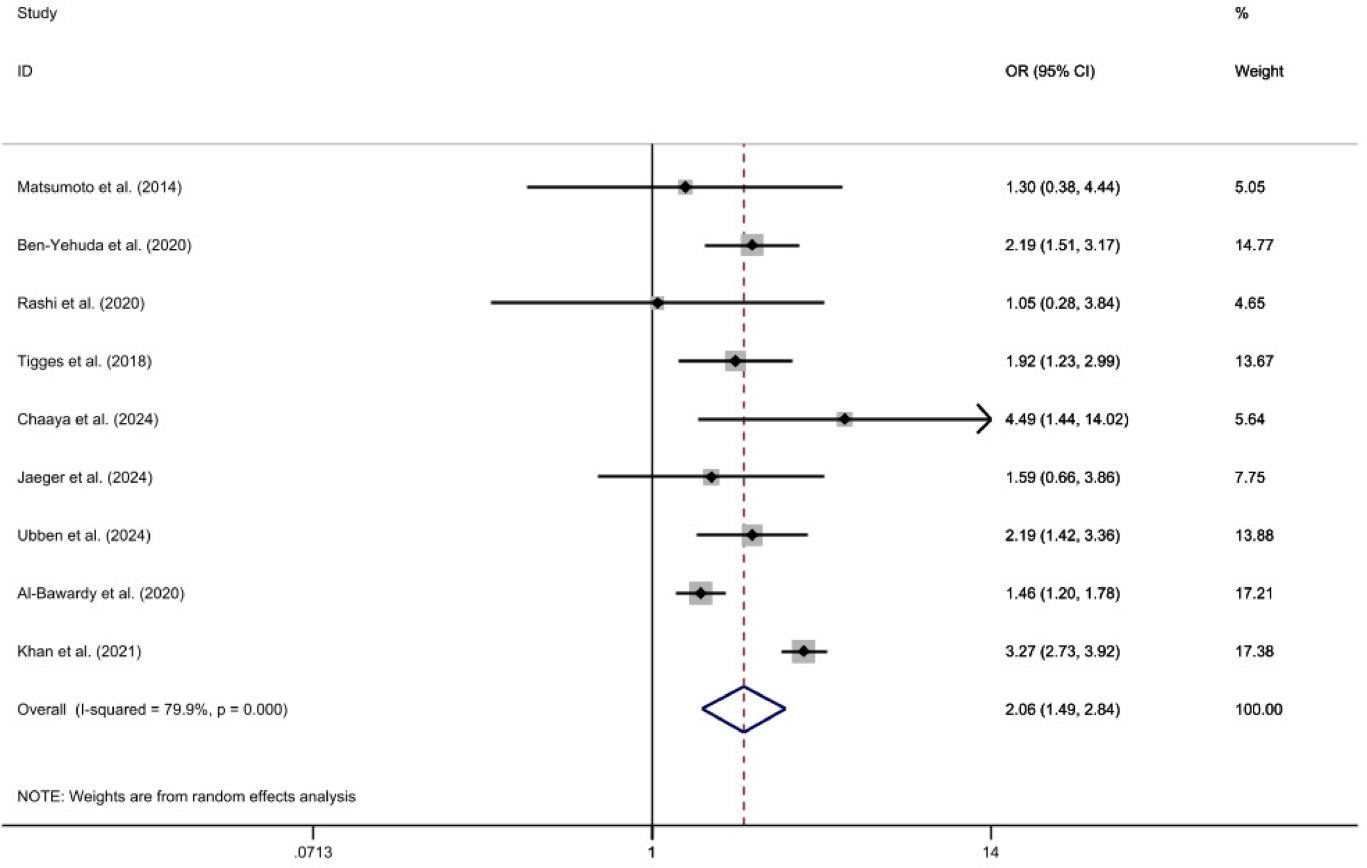
2-year All-cause mortality forest plot analysis.

Nine studies reported on the 2-year all-cause mortality rate (23–32). The heterogeneity tests yielded an *I*^2^ of 79.9%, indicating significant heterogeneity among the studies. Consequently, a random-effects model was employed for the meta-analysis. The results showed that the 2-year all-cause mortality rate in the PH group was significantly higher compared to the Non-PH group, with a statistically significant difference [*OR* = 2.06, 95%CI (1.49, 2.84), *p* < *0.01*, *I*^2^ = 79.9%]. Refer to the accompanying Figure 2.

### 4.2 Secondary outcomes

#### 4.2.1 Hospitalization for heart failure

Five studies reported on the heart failure rehospitalization rate (25, 27–29, 32). The heterogeneity assessment revealed an *I*^2^ of 41.7%, so a fixed-effect model was used for the meta-analysis. The results indicated that the heart failure rehospitalization rate was significantly higher in the PH group compared to the Non-PH group, with statistically significant differences [*OR* = 1.56, 95%CI (1.29, 1.76), *p* < 0.01, *I*^2^ = 41.7%]. Refer to Table 3.

**Table 3:** Secondary outcomes.

Outcomes	Studies	Heterogeneity test result		Effect model	Meta-analysis results	
		*p* value	*I*^2^(%)		*OR*(95%*CI*)	*p* value
30-day all-cause mortality	6 (23, 24, 27, 28, 31, 32)	0.927	0	Fixed	20.10 (1.78,2.47)	<0.01
TTE	2 (24, 27)	0.594	0	Fixed	2.09 (1.05,4.18)	0.04
RHC	2 (28, 32)	0.714	0	Fixed	2.47 (1.46,4.16)	<0.01
Hospitalization for heart failure	5 (25, 27–29, 32)	0.143	41.7	Fixed	1.56 (1.29,1.76)	<0.01
TTE	2 (25, 27)	0.327	0	Random	1.91 (1.44,2.53)	<0.01
RHC	3 (28, 29, 32)	0.067	63.1	Random	1.46 (1.22,1.76)	0.07
Mild or Moderate PH	3 (27, 29, 32)	0.433	0	Random	1.36 (1.13,1.62)	<0.01
Severe PH	3 (27, 29, 32)	0.043	68.3	Random	1.72 (1.37,2.16)	0.04
Cardiac death	4 (23, 25, 27, 32)	0.495	0	Fixed	2.00 (1.61,2.49)	<0.01
Length of stay	4 (23, 24, 28, 31)	0.826	0	Fixed	0.17 (0.14,0.20)	<0.01

TTE, transthoracic echocardiography; RHC, right heart floating catheter; PH, pulmonary hypertension; OR, Odds ratios; CI, confidence interval.

#### 4.2.2 Thirty-day all-cause mortality

Six studies reported on 30-day all-cause mortality rates (23, 24, 27, 28, 31, 32). The heterogeneity tests showed zero heterogeneity (*I*^2^ = 0%), leading to the use of a fixed-effects model for the meta-analysis. The results demonstrated a statistically significant increase in 30-day all-cause mortality in the PH group compared to the Non-PH group [*OR* = 2.10, 95%CI (1.78, 2.47), *p* < 0.01, *I*^2^ = 0%] (see Table 3).

#### 4.2.3 Cardiac death

Four studies reported on cardiac mortality outcomes (23, 25, 27, 32). The heterogeneity test indicated *I*^2^ = 0%, leading to the use of a fixed effect model for the meta-analysis. The results revealed that cardiac mortality was significantly higher in the PH group compared to the Non-PH group, with a statistically significant difference [*OR* = 2.00, 95%CI (1.61, 2.49), *p* < 0.01, *I*^2^ = 0%]; see Table 3 for details.

#### 4.2.4 Length of stay

Four studies reported on the length of hospital stay (23, 24, 28, 31). Heterogeneity testing showed *I*^2^ = 0%, indicating no observed heterogeneity, so a fixed-effect model was used for the meta-analysis. The results indicated a significantly shorter hospital stay for the PH group compared to the Non-PH group, with a statistically significant difference [*OR* = 0.17, 95%CI (0.14, 0.20), *p* < 0.01, *I*^2^ = 0%], as shown in Table 3.

### 4.3 Subgroup analysis

Given the high heterogeneity observed in some outcome indicators, subgroup analyses were performed to explore the sources of heterogeneity. The analysis focused on three aspects: the severity of patient PH, the diagnostic criteria for PH, and the methods used for PH examination. For instance, the results of the subgroup analysis for the 2-year all-cause mortality rate are detailed below. The results for other outcome indicators can be found in Table 3.

#### 4.3.1 Two-year all-cause mortality subgroup analysis by severity of PH

**Figure 3:**
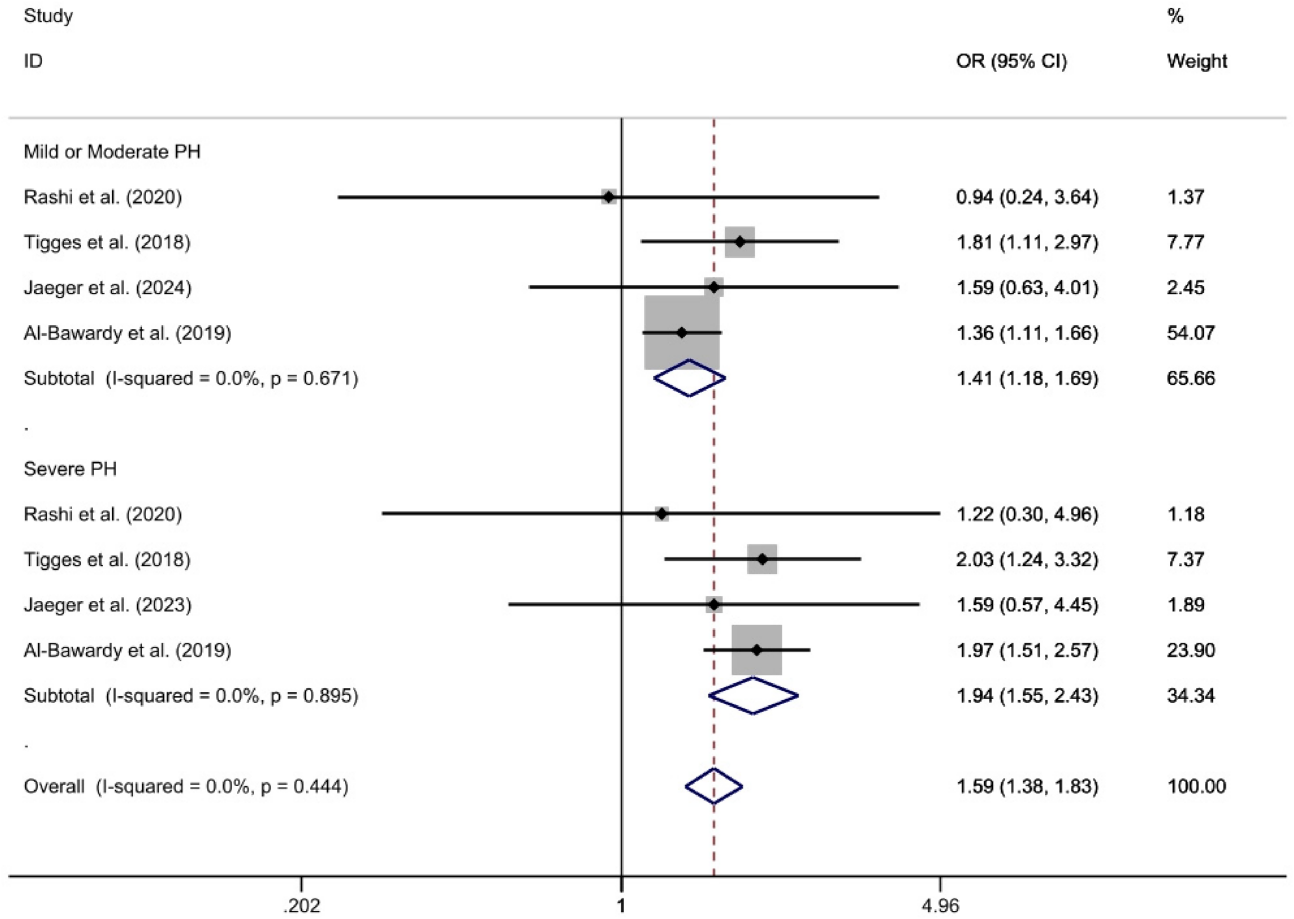
For the subgroup analysis of observed 2-year all-cause mortality rates, a forest plot is utilized according to the severity of pulmonary hypertension.

The analysis divided groups based on the severity of PH into mild/moderate PH and severe PH subgroups. The mild/moderate PH subgroup, which included four studies (26, 27, 29, 32), was analyzed using a fixed-effect model. This analysis revealed that the PH group had a significantly higher 2-year all-cause mortality rate compared to the Non-PH group [*OR* = 1.41, 95%CI (1.18, 1.69), *p* < 0.01, *I*^2^ = 0%]. Similarly, the severe PH subgroup, also comprising four studies (26, 27, 29, 32), showed no observed heterogeneity (*I*^2^ = 0%) and demonstrated that the PH group had a significantly higher 2-year all-cause mortality rate compared to the non-PH group [*OR* = 1.94, 95%CI (1.55, 2.43), *p* < 0.01, *I*^2^ = 0%] (Figure 3).

#### 4.3.2 Subgroup analysis of 2-year all-cause mortality based on PH diagnostic criteria

**Figure 4:**
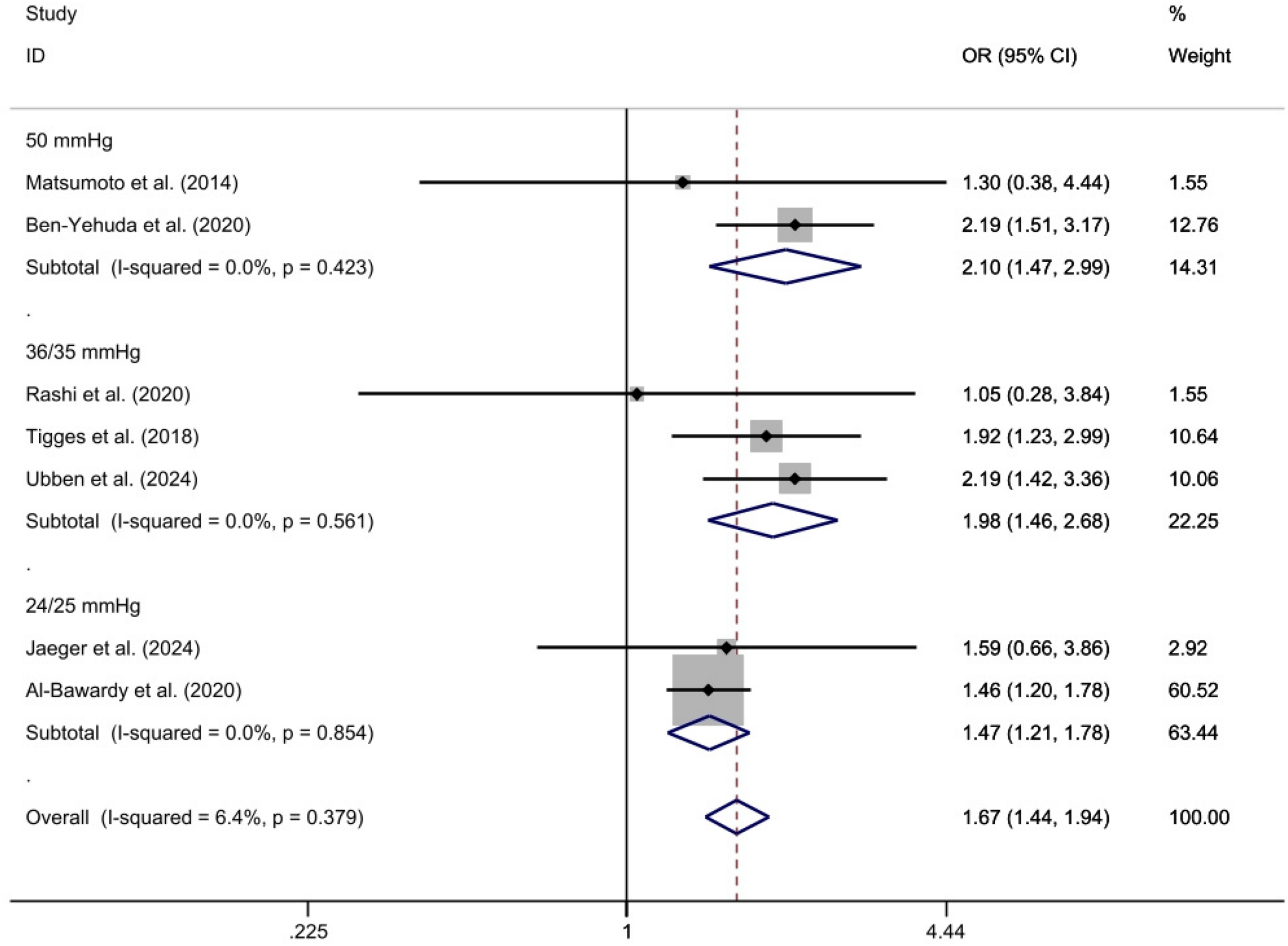
2-year All-cause mortality rate ratio subgroup analysis forest plot using the specified assessment method.

Subgroup analysis was conducted based on different diagnostic criteria for PH. The subgroups were defined by thresholds of 24/25 mm Hg, 35/36 mm Hg, and 50 mm Hg. The analysis include two studies for the 24/25 mm Hg subgroup (29, 32), three studies for the 35/36 mm Hg subgroup (26, 27, 30), and two studies for the 50 mm Hg subgroup (23, 25). Given that heterogeneity tests showed *I*^2^ = 0% for each subgroup, a fixed-effect model was applied. The results were as follows: for the 24/25 mm Hg subgroup [*OR* = 1.47, 95%CI (1.21, 1.78), *p* < 0.01, *I*^2^ = 0%]; for the 35/36 mm Hg subgroup [*OR* = 1.98, 95%*CI* (1.46, 2.68), *P* < 0.01, *I*^2^ = 0%]; and for the 50 mm Hg subgroup [*OR* = 2.10, 95%CI (1.47, 2.99), *p* < 0.01, *I*^2^ = 0%]. Statistical significance was observed in the odds ratios between the PH and Non-PH groups across all diagnostic criteria subgroups. These odds ratios and confidence intervals reflect the likelihood of all-cause mortality in each subgroup compared to the control group (Figure 4).

#### 4.3.3 Subgroup analysis of 2-year all-cause mortality rates in PH vs. non-PH groups

**Figure 5:**
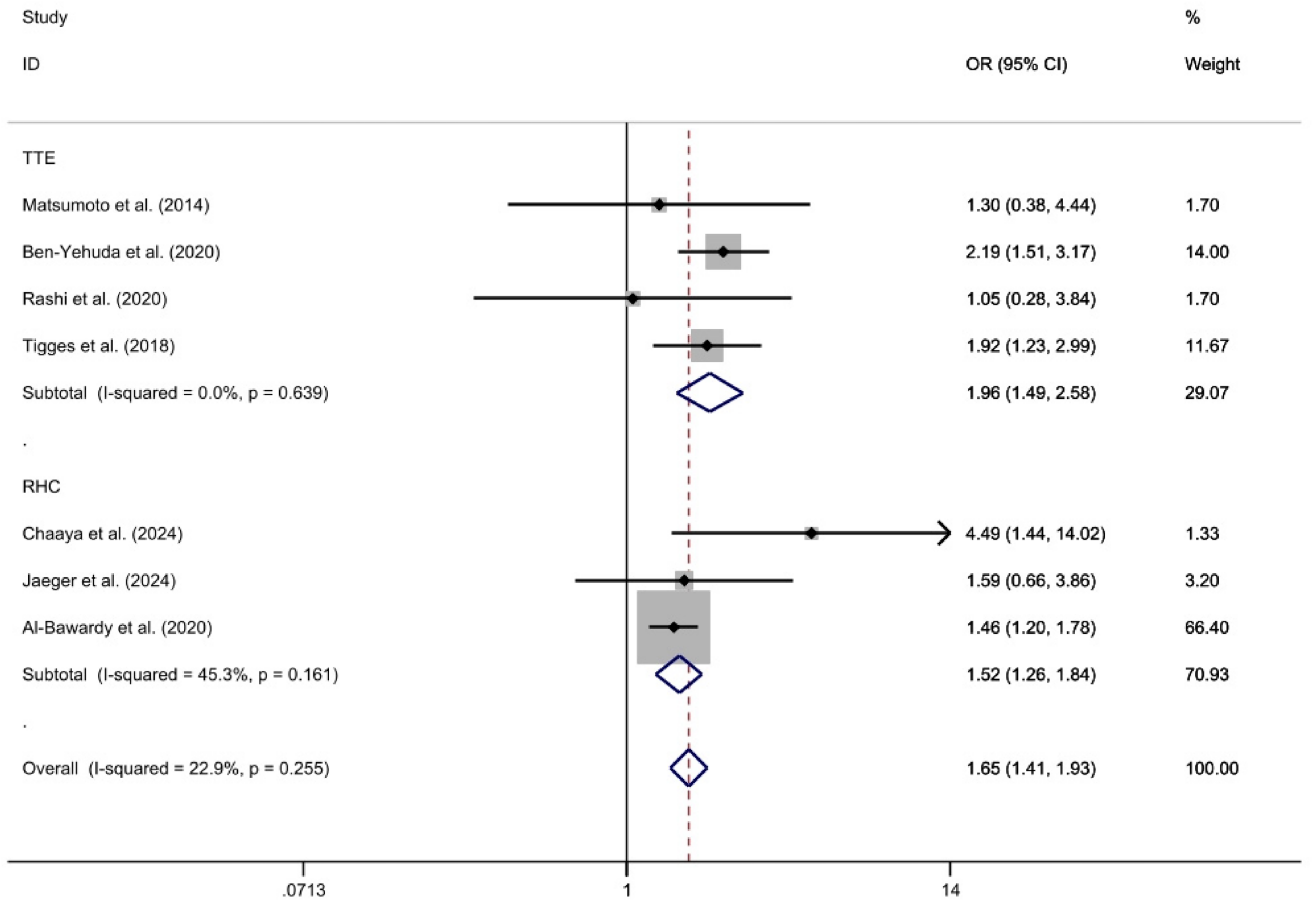
Forest plot illustrating subgroup analysis according to diagnostic criteria for 2-year mortality rates in a medical study.

The TTE subgroup, which included four studies (23, 25–27), was analyzed using a fixed-effect model. This analysis indicated that the 2-year all-cause mortality rate was significantly higher in the PH group compared to the Non-PH group [*OR* = 1.96, 95%CI (1.49, 2.58), *p* < 0.01, *I*^2^ = 0%]. Similarly, the RHC subgroup, consisting of three studies (28, 29, 32), was also analyzed using a fixed-effect model. This analysis revealed that the 2-year all-cause mortality rate was significantly higher in the PH group compared to the Non-PH group [*OR* = 1.52, 95%CI (1.26, 1.84), *p* < 0.01, *I*^2^ = 45.3%]. Both analyses demonstrated statistically significant differences in mortality rates between the PH and Non-PH groups (Figure 5).

### 4.4 Sensitivity analysis

**Figure 6:**
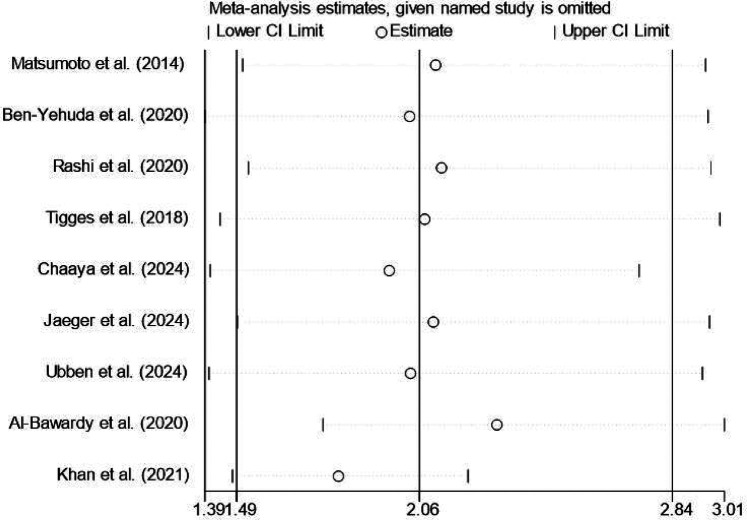
Two-year sensitivity analysis for All-cause mortality rates.

Sensitivity analysis was performed using sequentially excluding individual studies to test the robustness of the studies. For instance, when examine the 2-year all-cause mortality rate, no significant variations were observed, indicating that the results were robust and reliable (Figure 6).

### 4.5 Publication bias

**Figure 7:**
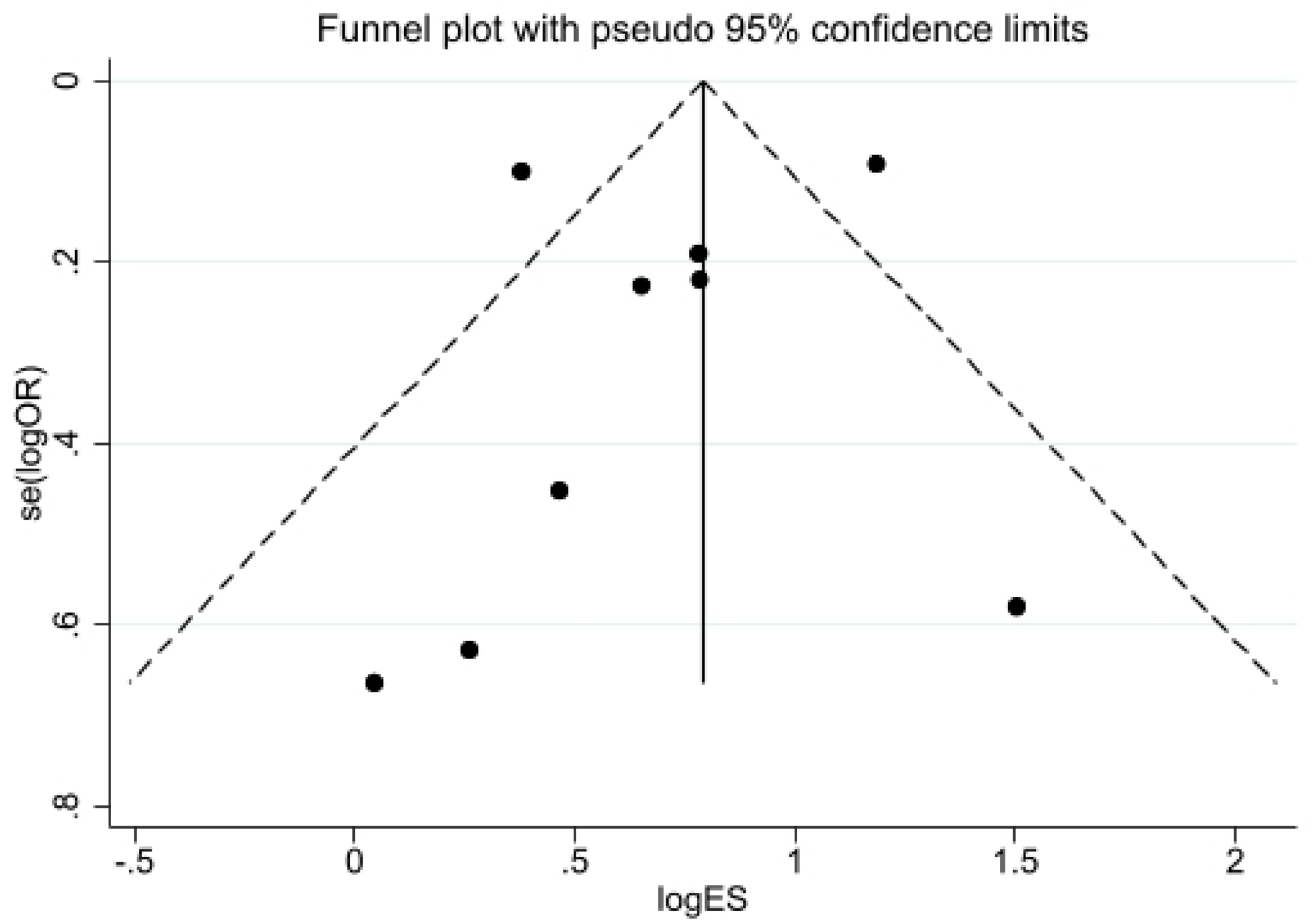
2-year All-cause mortality funnel chart.

**Figure 8:**
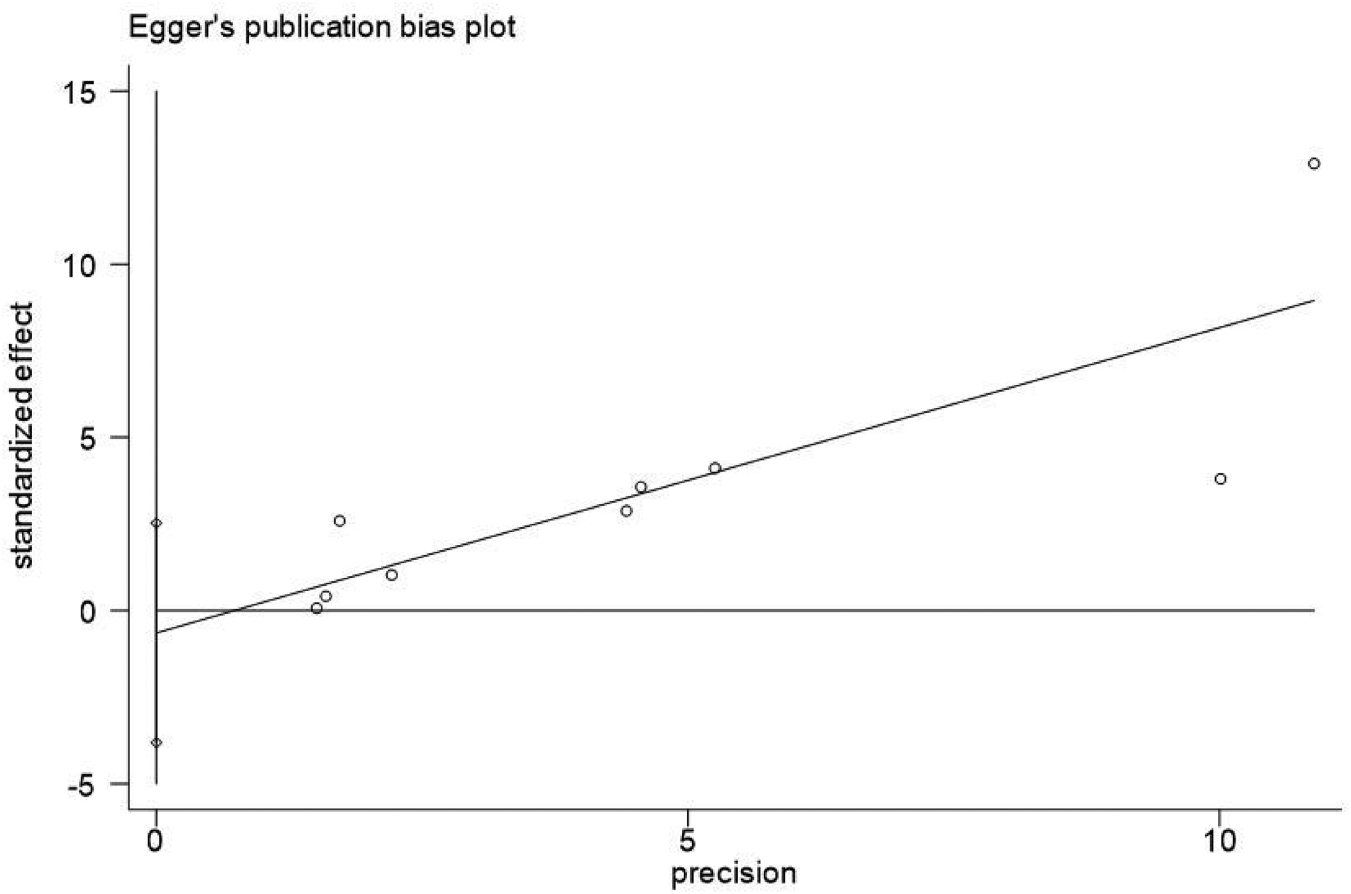
Application of egger's test to the 2-year All-cause mortality rate.

To assess publication bias, funnel plots were created using Stata 17.0 software, and Egger's test was conducted. The funnel plots for all outcome indicators did not show no significant asymmetry. For example, the funnel plot for the 2-year all-cause mortality rate is provided. Results from Egger's test were as follows: 30-day all-cause mortality rate (*p* = 0.553), 2-year all-cause mortality rate (*p* = 0.645), cardiogenic mortality rate (*p* = 0.152), rehospitalization rate for heart failure (*p* = 0.151), and length of hospital stay (*p* = 0.245). The results indicated that there was no significant publication bias across any of the outcome index (Figures 7, 8). Furthermore, the quality assessment of evidence for each outcome indicator also indicates that the quality of our research evidence is relatively high, as shown in Table 4.

**Table 4:** GRADE evidence level assessment.

Certainty assessment							No of patients	Certainty
No of studies	Study design	Risk of bias	Inconsistency	Indirectness	Imprecision	Publication bias		
30-day all-cause mortality								
6	Cohort study	Not serious	Not serious	Not serious	Not serious	Undetected	27,461	Moderate
2-years all-cause mortality								
9	Cohort study	Not serious	Not serious	Not serious	Not serious	Undetected	27,816	Moderate
Hospitalization for heart failure								
5	Cohort study	Not serious	Not serious	Not serious	Not serious	Undetected	5,594	Moderate
Cardiac death								
4	Cohort study	Not serious	Not serious	Not serious	Not serious	Undetected	5,333	Moderate
Length of stay								
4	Cohort study	Not serious	Not serious	Not serious	serious	Undetected	22,747	Low

## 5 Discussion

The discussion section analyzes the impact of PH on the prognosis of patients undergoing TEER for moderate to severe MR. This study involved 28,404 patients across 10 studies. The analysis results are as follows: (1) Patients with MR who also had PH had significantly higher 30-day and 2-year all-cause mortality rates post-TEER compared to patients without PH. (2) Preoperative PH was associated with higher postoperative cardiac mortality rates, increased rates of rehospitalization due to heart failure, and longer hospital stays. (3) Subgroup analysis revealed that the 2-year all-cause mortality rate and rate of rehospitalization due to heart failure increased with the severity of PH. This increase may be attributed to heterogeneity in the methods of PH measurement, PH diagnostic criteria, and severity of PH.

TEER is increasingly chosen by clinicians as a minimally invasive and safe treatment option for moderate to severe MR (33–36). Given this, it is necessary to understand how PH impacts the prognosis of patients with MR undergoing TEER. In 2021, Khan et al. (24) conducted a study using data from the National Inpatient Sample (NIS) database spanning January 2014 to December 2018, which included 21,505 inpatient samples. They found that patients with PH had a significantly higher 2-year all-cause mortality rate compared to those without PH (OR = 3.27, *p* < 0.01). Additionally, the length of hospital stay for patients with PH was longer than for those without PH (6.1 days vs. 4.5 days, *p* < 0.01), both of which were statistically significant. However, Ahmed et al. (31) conducted a study in 2019 using the National Inpatient Sample (NIS), including 1,037 patients who underwent TEER from 2011 to 2015, with a PH prevalence of 32.6%. This study found no statistically significant difference in in-hospital mortality rates between the PH group and non-PH groups (3.2% vs. 2.1%, *OR* = 1.57, *p* = 0.335), nor in the lengths of hospital stay (*OR* = 1.13, *p* = 0.316). The study also examined other complications, including bleeding requiring transfusion, with an incidence of 8.5% in the PH group vs. 7.2% in the non-PH group *(OR* = 1.17, *p* = 0.587), cardiogenic shock (4.4% vs. 4.5%, *OR* = 0.98, *p* = 0.951), acute respiratory failure (15.2% vs. 13.1%, *OR* = 1.23, *p* = 0.460), postoperative sepsis (2.75% vs. 3.9%, *OR* = 0.66, *p* = 0.340), and postoperative deep vein thrombosis or pulmonary embolism (2.7% vs. 3.9%, *OR* = 1.98, *p* = 0.348). None of these differences were statistically significant, leading to the conclusion that PH does not adversely affect patients undergoing TEER. Given the inconsistency findings, our study aimed to provide a comprehensive meta-analysis to determine the precise impact of PH on outcomes for patients undergoing TEER.

For the 2-year all-cause mortality, Chaaya Khan et al. (28) reported that PH significantly increased the 2-year all-cause mortality rate (11.4% vs. 36.7%, *p* = 0.006), while the 2020 study by Rashi et al. (26) found no statistical difference in the 2-year all-cause mortality rate between the PH and the non-PH groups (*OR* = 1.05, *p* > 0.05). Regarding the 30-day all-cause mortality rate, Ahmed et al. (31) reported that PH had almost no impact (*OR* = 1.57, *p* > 0.05), whereas Matsumoto et al. (23) found the opposite (*OR* = 4.68, *p* < 0.01). In our study, PH was associated with a significant increase in postoperative mortality rates both in the long term (2 years, *OR* = 2.06, *p* < 0.01) and short term (30 days, *OR* = 2.10, *p* < 0.01), consistent with the previous research. Possible contributing factors include: (1) PH increases the complexity and risk of TEER. (2) PH increases the afterload on the right ventricle, potentially leading to deterioration of right heart function and exacerbating the patient's condition over time. (3) PH makes left atrial ejection more difficult, raising pulmonary venous pressure, which increases the risk of pulmonary edema and exacerbates heart failure. (4) Although TEER reduces MR, a rapid decrease in preload in patients with concurrent PH may reduce cardiac output, causing hemodynamic instability and potentially triggering complications, thereby increasing the risk of death (37, 38). Regarding cardiogenic mortality, Matsumoto et al. (23) found that the PH group had a lower cardiogenic mortality rate than the non-PH group (*OR* = 0.88, *p* > 0.05), while our study found the opposite (OR = 2.01, *p* < 0.01), which aligns with findings from other studies. Additionally, our findings suggest that PH has a negative impact on heart failure readmission rates and lengths of hospital stays, consistent with previous studies. The mechanisms underlying these outcomes may include increased right ventricular pressure load and pulmonary vascular resistance caused by PH, which can lead to chronic hypoxia, cardiac remodeling, arrhythmias, and a systemic inflammatory response. Collectively, these factors together contribute to a significant increase in the 30-day and 2-year all-cause mortality rates, heart failure readmission rates, cardiogenic mortality, and hospital stay duration (39–42). In our meta-analysis, we performed subgroup analyses on highly heterogeneous outcome indicators and found that the heterogeneity primarily stemmed from PH measurement methods, PH diagnostic criteria, and PH severity. Therefore, it is crucial to develop appropriate prevention and treatment strategies for patients with moderate to severe MR with concurrent PH to improve their prognosis.

## 6 Limitations

(1) This study included only English-language scientific literature, which may introduce potential selection bias. (2) The included studies were exclusively cohort studies, without randomized controlled trials (RCTs), which may affect the reliability of the research findings. (3) The study did not further explore different types of MR, such as ischemic and non-ischemic. (4) Some studies had small patient sample sizes, potentially diminishing their reference value. (5) The study did not categorize PH into pre-capillary, isolated post-capillary, and combined pre- and post-capillary PH for analysis.

## 7 Conclusion

In patients with moderate to severe MR, PH is associated with increased all-cause mortality, heart failure readmission rates, and cardiogenic mortality at both 30 days and 2 years post-TEER. Therefore, patients with moderate to severe MR undergoing TEER with concurrent PH require more comprehensive treatment strategies to improve their prognosis.

## Data Availability

The raw data supporting the conclusions of this article will be made available by the authors, without undue reservation.
