# Supplementary material for: The impact of pulmonary hypertension on prognosis in moderate-to-severe mitral regurgitation patients treated with transcatheter edge-to-edge mitral valve repair: a comprehensive meta-analysis
Source: Front Cardiovasc Med. 2025 Jan 10;11:1489674. doi: 10.3389/fcvm.2024.1489674 (PMC11757250; doi:10.3389/fcvm.2024.1489674)
Supplement: Supplementary file 1 [file Datasheet1.zip › Embase.docx]

## Embase

**#10**

#3 AND #6 AND #9

[380](https://www-embase-com.uic.sjlib.cn/)

**#9**

#7 OR #8

[140,265](https://www-embase-com.uic.sjlib.cn/)

**#8**

**'essential pulmonary hypertension'** OR **'hypertension, lung'** OR **'hypertension, pulmonary'** OR **'hypertensive pulmonary vascular disease'** OR **'idiopathic pulmonary arterial hypertension'** OR **'lung arterial hypertension'** OR **'lung artery hypertension'** OR **'lung hypertension'** OR **'primary pulmonary hypertension'** OR **'pulmonary artery hypertension'** OR **'pulmonary fixed hypertension'** OR **'pulmonary hypertensive disease'** OR **'pulmonary hypertensive diseases'** OR **'pulmonary hypertensive disorder'** OR **'pulmonary hypertensive disorders'** OR **'pulmonary hypertension'** OR **'familial primary pulmonary hypertension'** OR **'persistent fetal circulation syndrome'** OR **'pulmonary arterial hypertension'**

[131,279](https://www-embase-com.uic.sjlib.cn/)

**#7**

**'pulmonary hypertension'**/exp

[127,369](https://www-embase-com.uic.sjlib.cn/)

**#6**

#4 OR #5

[66,152](https://www-embase-com.uic.sjlib.cn/)

**#5**

**'insufficiency, mitral valve'** OR **'valve insufficiency, mitral'** OR **'incompetence, mitral'** OR **'insufficiency, mitral'** OR **'regurgitation, mitral'** OR **'bicuspid cardiac valve incompetence'** OR **'bicuspid cardiac valve insufficiency'** OR **'bicuspid cardiac valve regurgitation'** OR **'bicuspid heart valve incompetence'** OR **'bicuspid heart valve insufficiency'** OR **'bicuspid heart valve regurgitation'** OR **'bicuspid incompetence'** OR **'bicuspid insufficiency'** OR **'bicuspid regurgitation'** OR **'bicuspid valve insufficiency'** OR **'bicuspid valve regurgitation'** OR **'bicuspid valvular incompetence'** OR **'bicuspid valvular insufficiency'** OR **'bicuspid valvular regurgitation'** OR **'heart valve incompetence, mitral'** OR **'heart valve insufficiency, mitral'** OR **'heart valve regurgitation, mitral'** OR **'incompetence, mitral valve'** OR **'left atrioventricular cardiac valve incompetence'** OR **'left atrioventricular cardiac valve insufficiency'** OR **'left atrioventricular cardiac valve regurgitation'** OR **'left atrioventricular cardiac valvular incompetence'** OR **'left atrioventricular heart valve incompetence'** OR **'left atrioventricular heart valve insufficiency'** OR **'left atrioventricular heart valve regurgitation'** OR **'left atrioventricular incompetence'** OR **'left atrioventricular insufficiency'** OR **'left atrioventricular regurgitation'** OR **'left atrioventricular valve incompetence'** OR **'left atrioventricular valve insufficiency'** OR **'left atrioventricular valve regurgitation'** OR **'mitral cardiac valve incompetence'** OR **'mitral cardiac valve insufficiency'** OR **'mitral cardiac valve regurgitation'** OR **'mitral heart valve incompetence'** OR **'mitral heart valve insufficiency'** OR **'mitral heart valve regurgitation'** OR **'mitral incompetence'** OR **'mitral insufficiency'** OR **'mitral paravalvular regurgitation'** OR **'mitral regurgitation'** OR **'mitral valve incompetence'** OR **'mitral valve insufficiency'** OR **'mitral valvular incompetence'** OR **'mitral valvular insufficiency'** OR **'mitral valvular regurgitation'** OR **'mitralis regurgitation'** OR **'regurgitation, mitral valve'** OR **'valve incompetence, mitral'** OR **'valve regurgitation, mitral'** OR **'mitral valve regurgitation'**

[66,152](https://www-embase-com.uic.sjlib.cn/)

**#4**

**'mitral valve regurgitation'**/exp

[60,776](https://www-embase-com.uic.sjlib.cn/)

#3

#1 OR #2

[72,674](https://www-embase-com.uic.sjlib.cn/)*

**#2**

**'edge-to-edge transcatheter mitral valve repair'** OR **'mitral valve transcatheter edge-to-edge repair'** OR **'transcatheter edge-to-edge mitral valve repair'** OR **'transcatheter mitral valve edge-to-edge repair'** OR **'transcatheter edge to edge mitral valve repair'** OR **'teer'** OR **'tmvr'** OR **'mitraclip'** OR **'mitral clip'** OR **'mitral valve clip'** OR **'pascal'**

[78,056](https://www-embase-com.uic.sjlib.cn/)

**#1**

'edge to edge mitral valve repair'/exp

1,252
