# Supplementary material for: The impact of pulmonary hypertension on prognosis in moderate-to-severe mitral regurgitation patients treated with transcatheter edge-to-edge mitral valve repair: a comprehensive meta-analysis
Source: Front Cardiovasc Med. 2025 Jan 10;11:1489674. doi: 10.3389/fcvm.2024.1489674 (PMC11757250; doi:10.3389/fcvm.2024.1489674)
Supplement: Supplementary file 1 [file Datasheet1.zip › Cochrane Library.docx]

## Cochrane Library

Search Name:

Date Run: 29/07/2024 10:22:13

Comment:

ID Search Hits

#1 transcatheter edge to edge mitral valve repair 84

#2 (transcatheter edge-to-edge repair):ti,ab,kw OR (mitral valve transcatheter edge-to-edge repair (teer)):ti,ab,kw OR (transcatheter edge-to-edge mitral valve repair (tmvr)):ti,ab,kw OR (transcatheter mitral valve edge-to-edge repair):ti,ab,kw OR (transcatheter edge to edge mitredge-to-edge transcatheter mitral valve repair):ti,ab,kw (Word variations have been searched) 92

#3 (mitral valve al valve repair):ti,ab,kw OR (mitral valve clip):ti,ab,kw OR (MitraClip):ti,ab,kw OR (mitral clip):ti,ab,kw OR (mitral valve clip):ti,ab,kw (Word variations have been searched) 240

#4 Pascal 468

#5 #1 OR #2 OR #3 OR #4 969

#6 MeSH descriptor: [Mitral Valve Insufficiency] explode all trees 600

#7 (Insufficiency, Mitral Valve):ti,ab,kw OR (Valve Insufficiency, Mitral):ti,ab,kw OR (Mitral Incompetence):ti,ab,kw OR (Incompetence, Mitral):ti,ab,kw OR (Mitral Insufficiency):ti,ab,kw (Word variations have been searched) 806

#8 (Insufficiency, Mitral):ti,ab,kw OR (Mitral Regurgitation):ti,ab,kw OR (Regurgitation, Mitral):ti,ab,kw OR (Mitral Valve Incompetence):ti,ab,kw OR (Incompetence, Mitral Valve):ti,ab,kw (Word variations have been searched) 1485

#9 (Valve Incompetence, Mitral):ti,ab,kw OR (Mitral Valve Regurgitation):ti,ab,kw OR (Regurgitation, Mitral Valve):ti,ab,kw OR (Valve Regurgitation, Mitral):ti,ab,kw OR (bicuspid cardiac valve incompetence):ti,ab,kw (Word variations have been searched) 1117

#10 (bicuspid cardiac valve insufficiency):ti,ab,kw OR (bicuspid cardiac valve regurgitation):ti,ab,kw OR (bicuspid heart valve incompetence):ti,ab,kw OR (bicuspid heart valve insufficiency):ti,ab,kw OR (bicuspid heart valve regurgitation):ti,ab,kw (Word variations have been searched) 29

#11 (bicuspid incompetence):ti,ab,kw OR (bicuspid insufficiency):ti,ab,kw OR (bicuspid regurgitation):ti,ab,kw OR (bicuspid valve insufficiency):ti,ab,kw OR (bicuspid valve regurgitation):ti,ab,kw (Word variations have been searched) 44

#12 (bicuspid valvular incompetence):ti,ab,kw OR (bicuspid valvular insufficiency):ti,ab,kw OR (bicuspid valvular regurgitation):ti,ab,kw OR (heart valve incompetence, mitral):ti,ab,kw OR (heart valve insufficiency, mitral):ti,ab,kw (Word variations have been searched) 616

#13 (heart valve regurgitation, mitral):ti,ab,kw OR (incompetence, mitral valve):ti,ab,kw OR (left atrioventricular cardiac valve incompetence):ti,ab,kw OR (left atrioventricular cardiac valve insufficiency):ti,ab,kw OR (left atrioventricular cardiac valve regurgitation):ti,ab,kw (Word variations have been searched) 954

#14 (left atrioventricular cardiac valvular incompetence):ti,ab,kw OR (left atrioventricular heart valve incompetence):ti,ab,kw OR (left atrioventricular heart valve insufficiency):ti,ab,kw OR (left atrioventricular heart valve regurgitation):ti,ab,kw OR (left atrioventricular incompetence):ti,ab,kw (Word variations have been searched) 48

#15 (left atrioventricular insufficiency):ti,ab,kw OR (left atrioventricular regurgitation):ti,ab,kw OR (left atrioventricular valve incompetence):ti,ab,kw OR (left atrioventricular valve insufficiency):ti,ab,kw OR (left atrioventricular valve regurgitation):ti,ab,kw (Word variations have been searched) 74

#16 (mitral cardiac valve incompetence):ti,ab,kw OR (mitral cardiac valve insufficiency):ti,ab,kw OR (mitral cardiac valve regurgitation):ti,ab,kw OR (mitral heart valve incompetence):ti,ab,kw OR (mitral heart valve insufficiency):ti,ab,kw (Word variations have been searched) 896

#17 (mitral heart valve regurgitation):ti,ab,kw OR (mitral incompetence):ti,ab,kw OR (mitral insufficiency):ti,ab,kw OR (mitral paravalvular regurgitation):ti,ab,kw OR (mitral regurgitation):ti,ab,kw (Word variations have been searched) 1487

#18 (mitral valve incompetence):ti,ab,kw OR (mitral valve insufficiency):ti,ab,kw OR (mitral valvular incompetence):ti,ab,kw OR (mitral valvular insufficiency):ti,ab,kw OR (mitral valvular regurgitation):ti,ab,kw (Word variations have been searched) 843

#19 (mitralis regurgitation):ti,ab,kw OR (regurgitation, mitral valve):ti,ab,kw OR (valve incompetence, mitral):ti,ab,kw OR (valve regurgitation, mitral):ti,ab,kw OR (mitral valve regurgitation):ti,ab,kw (Word variations have been searched) 1117

#20 #6 OR #7 OR #8 OR #9 OR #10 OR #11 OR #12 OR #13 OR #14 OR #15 OR #16 OR #17 OR #18 OR #19 1567

#21 MeSH descriptor: [Hypertension, Pulmonary] explode all trees 1689

#22 (essential pulmonary hypertension):ti,ab,kw OR (familial primary pulmonary hypertension):ti,ab,kw OR (hypertension, lung):ti,ab,kw OR (hypertension, pulmonary):ti,ab,kw OR (hypertensive pulmonary vascular disease):ti,ab,kw (Word variations have been searched) 8554

#23 (idiopathic pulmonary arterial hypertension):ti,ab,kw OR (lung arterial hypertension):ti,ab,kw OR (lung artery hypertension):ti,ab,kw OR (lung hypertension):ti,ab,kw OR (primary pulmonary hypertension):ti,ab,kw (Word variations have been searched) 6356

#24 (pulmonary arterial hypertension):ti,ab,kw OR (pulmonary artery hypertension):ti,ab,kw OR (pulmonary fixed hypertension):ti,ab,kw OR (pulmonary hypertensive disease):ti,ab,kw OR (pulmonary hypertensive diseases):ti,ab,kw (Word variations have been searched) 5375

#25 (pulmonary hypertensive disorder):ti,ab,kw OR (pulmonary hypertensive disorders):ti,ab,kw OR (pulmonary hypertension):ti,ab,kw OR (Familial Primary Pulmonary Hypertension):ti,ab,kw OR (Persistent Fetal Circulation Syndrome):ti,ab,kw (Word variations have been searched) 6515

#26 (Pulmonary Arterial Hypertension):ti,ab,kw OR (pulmonary hypertension):ti,ab,kw (Word variations have been searched) 6504

#27 #21 OR #22 OR #23 OR #24 OR #25 OR #26 8565

#28 #5 AND #20 AND #27 in Cochrane Reviews, Cochrane Protocols, Trials, Clinical Answers, Editorials, Special Collections 13
