# Supplementary material for: The impact of pulmonary hypertension on prognosis in moderate-to-severe mitral regurgitation patients treated with transcatheter edge-to-edge mitral valve repair: a comprehensive meta-analysis
Source: Front Cardiovasc Med. 2025 Jan 10;11:1489674. doi: 10.3389/fcvm.2024.1489674 (PMC11757250; doi:10.3389/fcvm.2024.1489674)
Supplement: Supplementary file 1 [file Datasheet1.zip › WOS.docx]

## Web of Science

10

**#3 AND #6 AND #9**

[52](https://www.webofscience.com/wos/woscc/summary/1a632d98-2897-447b-ad2a-56ac6c48b6d0-fed5f8e2/relevance/1)

9

**#7 OR #8**

[56,456](https://www.webofscience.com/wos/woscc/summary/2b7a5b9f-dc1f-48c8-8d99-27649399fe13-fed42ee6/relevance/1)

8

**AB=(essential pulmonary hypertension OR familial primary pulmonary hypertension OR hypertension, lung OR hypertension, pulmonary OR hypertensive pulmonary vascular disease OR idiopathic pulmonary arterial hypertension OR lung arterial hypertension OR lung artery hypertension OR lung hypertension OR primary pulmonary hypertension OR pulmonary arterial hypertension OR pulmonary artery hypertension OR pulmonary fixed hypertension OR pulmonary hypertensive disease OR pulmonary hypertensive diseases OR pulmonary hypertensive disorder OR pulmonary hypertensive disorders OR pulmonary hypertension OR Familial Primary Pulmonary Hypertension OR Persistent Fetal Circulation Syndrome OR Pulmonary Arterial Hypertension OR pulmonary hypertension)**

[56,456](https://www.webofscience.com/wos/woscc/summary/8f9d7324-d9b1-4817-a408-f53848926d14-fed42ed2/relevance/1)

7

**AB=(Hypertension, Pulmonary)**

[51,572](https://www.webofscience.com/wos/woscc/summary/a19d26da-0c48-4662-9a58-1492fb837be2-fed1b206/relevance/1)

6

**#4 OR #5**

[20,981](https://www.webofscience.com/wos/woscc/summary/acb44636-20cd-4145-b723-a29eefac9586-fed1ad4e/relevance/1)

5

**AB=(Insufficiency, Mitral Valve OR Valve Insufficiency, Mitral OR Mitral Incompetence OR Incompetence, Mitral OR Mitral Insufficiency OR Insufficiency, Mitral OR Mitral Regurgitation OR Regurgitation, Mitral OR Mitral Valve Incompetence OR Incompetence, Mitral Valve OR Valve Incompetence, Mitral OR Mitral Valve Regurgitation OR Regurgitation, Mitral Valve OR Valve Regurgitation, Mitral OR bicuspid cardiac valve incompetence OR bicuspid cardiac valve insufficiency OR bicuspid cardiac valve regurgitation OR bicuspid heart valve incompetence OR bicuspid heart valve insufficiency OR bicuspid heart valve regurgitation OR bicuspid incompetence OR bicuspid insufficiency OR bicuspid regurgitation OR bicuspid valve insufficiency OR bicuspid valve regurgitation OR bicuspid valvular incompetence OR bicuspid valvular insufficiency OR bicuspid valvular regurgitation OR heart valve incompetence, mitral OR heart valve insufficiency, mitral OR heart valve regurgitation, mitral OR incompetence, mitral valve OR left atrioventricular cardiac valve incompetence OR left atrioventricular cardiac valve insufficiency OR left atrioventricular cardiac valve regurgitation OR left atrioventricular cardiac valvular incompetence OR left atrioventricular heart valve incompetence OR left atrioventricular heart valve insufficiency OR left atrioventricular heart valve regurgitation OR left atrioventricular incompetence OR left atrioventricular insufficiency OR left atrioventricular regurgitation OR left atrioventricular valve incompetence OR left atrioventricular valve insufficiency OR left atrioventricular valve regurgitation OR mitral cardiac valve incompetence OR mitral cardiac valve insufficiency OR mitral cardiac valve regurgitation OR mitral heart valve incompetence OR mitral heart valve insufficiency OR mitral heart valve regurgitation OR mitral incompetence OR mitral insufficiency OR mitral paravalvular regurgitation OR mitral regurgitation OR mitral valve incompetence OR mitral valve insufficiency OR mitral valvular incompetence OR mitral valvular insufficiency OR mitral valvular regurgitation OR mitralis regurgitation OR regurgitation, mitral valve OR valve incompetence, mitral OR valve regurgitation, mitral OR mitral valve regurgitation)**

[20,981](https://www.webofscience.com/wos/woscc/summary/a00d5595-2662-4e9a-9343-bd4b9b310e5d-fed1987a/relevance/1)

4

**AB=(Mitral Valve Insufficiency)**

[1,913](https://www.webofscience.com/wos/woscc/summary/f9e9a423-e341-4841-baa6-14c423dc7bd8-fed1823c/relevance/1)

3

**#2 OR #1**

[13,079](https://www.webofscience.com/wos/woscc/summary/7c2b33ca-2d41-4b45-838d-b03f94285a39-fed5f8b3/relevance/1)

2

**AB=(edge-to-edge transcatheter mitral valve repair OR mitral valve transcatheter edge-to-edge repair OR mitral valve transcatheter edge-to-edge repair OR transcatheter edge-to-edge mitral valve repair OR transcatheter mitral valve edge-to-edge repair OR transcatheter edge to edge mitral valve repair OR OR TEER OR TMVR mitral valve clip OR MitraClip OR mitral clip OR mitral valve clip OR Pascal)**

[13,079](https://www.webofscience.com/wos/woscc/summary/d46799bc-f91f-490d-be4a-6696cd45eb79-fed5f8a2/relevance/1)

1

**AB=(transcatheter edge to edge mitral valve repair)**
