# Supplementary material for: The impact of pulmonary hypertension on prognosis in moderate-to-severe mitral regurgitation patients treated with transcatheter edge-to-edge mitral valve repair: a comprehensive meta-analysis
Source: Front Cardiovasc Med. 2025 Jan 10;11:1489674. doi: 10.3389/fcvm.2024.1489674 (PMC11757250; doi:10.3389/fcvm.2024.1489674)
Supplement: Supplementary file 1 [file Datasheet1.zip › Pubmed.docx]

## PubMed

| #14 |  |  | Search: ****#3 AND #9 AND #13**** Sort by: ****Most Recent**** | [71](https://pubmed.ncbi.nlm.nih.gov/?term=%233+AND+%239+AND+%2313&sort=date&size=200) | 09:50:35 |
| --- | --- | --- | --- | --- | --- |
| #13 |  |  | Search: ****#11 OR #12**** Sort by: ****Most Recent**** | [7,171](https://pubmed.ncbi.nlm.nih.gov/?term=%2311+OR+%2312&sort=date&size=200) | 09:48:30 |
| #12 |  |  | Search: ****transcatheter edge to edge mitral valve repair[Title/Abstract]**** Sort by: ****Most Recent**** | [221](https://pubmed.ncbi.nlm.nih.gov/?term=transcatheter+edge+to+edge+mitral+valve+repair[Title/Abstract]&sort=date&size=200) | 09:47:16 |
| #11 |  |  | Search: ****（edge-to-edge transcatheter mitral valve repair[Title/Abstract]) OR (mitral valve transcatheter edge-to-edge repair[Title/Abstract]) OR (mitral valve transcatheter edge-to-edge repair[Title/Abstract]) OR (transcatheter edge-to-edge mitral valve repair[Title/Abstract]) OR (transcatheter mitral valve edge-to-edge repair[Title/Abstract]) OR (transcatheter edge to edge mitral valve repair[Title/Abstract]) OR (TEER[Title/Abstract]) OR (TMVR[Title/Abstract]) OR (mitral valve clip[Title/Abstract]) OR (MitraClip[Title/Abstract]) OR (mitral clip[Title/Abstract]) OR (mitral valve clip[Title/Abstract]) OR (Pascal[Title/Abstract])**** Sort by: ****Most Recent**** | [7,171](https://pubmed.ncbi.nlm.nih.gov/?term=%EF%BC%88edge-to-edge+transcatheter+mitral+valve+repair[Title/Abstract])+OR+(mitral+valve+transcatheter+edge-to-edge+repair[Title/Abstract])+OR+(mitral+valve+transcatheter+edge-to-edge+repair[Title/Abstract])+OR+(transcatheter+edge-to-edge+mitral+valve+repair[Title/Abstract])+OR+(transcatheter+mitral+valve+edge-to-edge+repair[Title/Abstract])+OR+(transcatheter+edge+to+edge+mitral+valve+repair[Title/Abstract])+OR+(mitral+valve+clip[Title/Abstract])+OR+(MitraClip[Title/Abstract])+OR+(mitral+clip[Title/Abstract])+OR+(mitral+valve+clip[Title/Abstract])+OR+(Pascal[Title/Abstract])+OR+(TEER[Title/Abstract])+OR+(TMVR[Title/Abstract])&sort=date&size=200) | 09:46:03 |
| #9 |  |  | Search: ****#7 OR #8**** Sort by: ****Most Recent**** | [67,075](https://pubmed.ncbi.nlm.nih.gov/?term=%237+OR+%238&sort=date&size=200) | 08:22:33 |
| #8 |  |  | Search: ****（essential pulmonary hypertension[Title/Abstract]) OR (familial primary pulmonary hypertension[Title/Abstract]) OR (hypertension, lung[Title/Abstract]) OR (hypertension, pulmonary[Title/Abstract]) OR (hypertensive pulmonary vascular disease[Title/Abstract]) OR (idiopathic pulmonary arterial hypertension[Title/Abstract]) OR (lung arterial hypertension[Title/Abstract]) OR (lung artery hypertension[Title/Abstract]) OR (lung hypertension[Title/Abstract]) OR (primary pulmonary hypertension[Title/Abstract]) OR (pulmonary arterial hypertension[Title/Abstract]) OR (pulmonary artery hypertension[Title/Abstract]) OR (pulmonary fixed hypertension[Title/Abstract]) OR (pulmonary hypertensive disease[Title/Abstract]) OR (pulmonary hypertensive diseases[Title/Abstract]) OR (pulmonary hypertensive disorder[Title/Abstract]) OR (pulmonary hypertensive disorders[Title/Abstract]) OR (pulmonary hypertension[Title/Abstract]) OR (Familial Primary Pulmonary Hypertension[Title/Abstract]) OR (Persistent Fetal Circulation Syndrome[Title/Abstract]) OR (Pulmonary Arterial Hypertension[Title/Abstract])**** Sort by: ****Most Recent**** | [58,815](https://pubmed.ncbi.nlm.nih.gov/?term=%EF%BC%88essential+pulmonary+hypertension[Title/Abstract])+OR+(familial+primary+pulmonary+hypertension[Title/Abstract])+OR+(hypertension,+lung[Title/Abstract])+OR+(hypertension,+pulmonary[Title/Abstract])+OR+(hypertensive+pulmonary+vascular+disease[Title/Abstract])+OR+(idiopathic+pulmonary+arterial+hypertension[Title/Abstract])+OR+(lung+arterial+hypertension[Title/Abstract])+OR+(lung+artery+hypertension[Title/Abstract])+OR+(lung+hypertension[Title/Abstract])+OR+(primary+pulmonary+hypertension[Title/Abstract])+OR+(pulmonary+arterial+hypertension[Title/Abstract])+OR+(pulmonary+artery+hypertension[Title/Abstract])+OR+(pulmonary+fixed+hypertension[Title/Abstract])+OR+(pulmonary+hypertensive+disease[Title/Abstract])+OR+(pulmonary+hypertensive+diseases[Title/Abstract])+OR+(pulmonary+hypertensive+disorder[Title/Abstract])+OR+(pulmonary+hypertensive+disorders[Title/Abstract])+OR+(pulmonary+hypertension[Title/Abstract])+OR+(Familial+Primary+Pulmonary+Hypertension[Title/Abstract])+OR+(Persistent+Fetal+Circulation+Syndrome[Title/Abstract])+OR+(Pulmonary+Arterial+Hypertension[Title/Abstract])&sort=date&size=200) | 08:21:34 |
| #7 |  |  | Search: ****"Hypertension, Pulmonary"[Mesh]**** Sort by: ****Most Recent**** | [43,651](https://pubmed.ncbi.nlm.nih.gov/?sort=date&term="Hypertension,+Pulmonary"[Mesh]&size=200) | 08:20:00 |
| #3 |  |  | Search: ****#1 OR #2**** Sort by: ****Most Recent**** | [42,162](https://pubmed.ncbi.nlm.nih.gov/?term=%231+OR+%232&sort=date&size=200) | 08:15:03 |
| #2 |  |  | Search: ****(Insufficiency, Mitral Valve[Title/Abstract]) OR (Valve Insufficiency, Mitral[Title/Abstract]) OR (Mitral Incompetence[Title/Abstract]) OR (Incompetence, Mitral[Title/Abstract]) OR (Mitral Insufficiency[Title/Abstract]) OR (Insufficiency, Mitral[Title/Abstract]) OR (Mitral Regurgitation[Title/Abstract]) OR (Regurgitation, Mitral[Title/Abstract]) OR (Mitral Valve Incompetence[Title/Abstract]) OR (Incompetence, Mitral Valve[Title/Abstract]) OR (Valve Incompetence, Mitral[Title/Abstract]) OR (Mitral Valve Regurgitation[Title/Abstract]) OR (Regurgitation, Mitral Valve[Title/Abstract]) OR (Valve Regurgitation, Mitral[Title/Abstract]) OR (bicuspid cardiac valve incompetence[Title/Abstract]) OR (bicuspid cardiac valve insufficiency[Title/Abstract]) OR (bicuspid cardiac valve regurgitation[Title/Abstract]) OR (bicuspid heart valve incompetence[Title/Abstract]) OR (bicuspid heart valve insufficiency[Title/Abstract]) OR (bicuspid heart valve regurgitation[Title/Abstract]) OR (bicuspid incompetence[Title/Abstract]) OR (bicuspid insufficiency[Title/Abstract]) OR (bicuspid regurgitation[Title/Abstract]) OR (bicuspid valve insufficiency[Title/Abstract]) OR (bicuspid valve regurgitation[Title/Abstract]) OR (bicuspid valvular incompetence[Title/Abstract]) OR (bicuspid valvular insufficiency[Title/Abstract]) OR (bicuspid valvular regurgitation[Title/Abstract]) OR (heart valve incompetence, mitral[Title/Abstract]) OR (heart valve insufficiency, mitral[Title/Abstract]) OR (heart valve regurgitation, mitral[Title/Abstract]) OR (incompetence, mitral valve[Title/Abstract]) OR (left atrioventricular cardiac valve incompetence[Title/Abstract]) OR (left atrioventricular cardiac valve insufficiency[Title/Abstract]) OR (left atrioventricular cardiac valve regurgitation[Title/Abstract]) OR (left atrioventricular cardiac valvular incompetence[Title/Abstract]) OR (left atrioventricular heart valve incompetence[Title/Abstract]) OR (left atrioventricular heart valve insufficiency[Title/Abstract]) OR (left atrioventricular heart valve regurgitation[Title/Abstract]) OR (left atrioventricular incompetence[Title/Abstract]) OR (left atrioventricular insufficiency[Title/Abstract]) OR (left atrioventricular regurgitation[Title/Abstract]) OR (left atrioventricular valve incompetence[Title/Abstract]) OR (left atrioventricular valve insufficiency[Title/Abstract]) OR (left atrioventricular valve regurgitation[Title/Abstract]) OR (mitral cardiac valve incompetence[Title/Abstract]) OR (mitral cardiac valve insufficiency[Title/Abstract]) OR (mitral cardiac valve regurgitation[Title/Abstract]) OR (mitral heart valve incompetence[Title/Abstract]) OR (mitral heart valve insufficiency[Title/Abstract]) OR (mitral heart valve regurgitation[Title/Abstract]) OR (mitral incompetence[Title/Abstract]) OR (mitral insufficiency[Title/Abstract]) OR (mitral paravalvular regurgitation[Title/Abstract]) OR (mitral regurgitation[Title/Abstract]) OR (mitral valve incompetence[Title/Abstract]) OR (mitral valve insufficiency[Title/Abstract]) OR (mitral valvular incompetence[Title/Abstract]) OR (mitral valvular insufficiency[Title/Abstract]) OR (mitral valvular regurgitation[Title/Abstract]) OR (mitralis regurgitation[Title/Abstract]) OR (regurgitation, mitral valve[Title/Abstract]) OR (valve incompetence, mitral[Title/Abstract]) OR (valve regurgitation, mitral[Title/Abstract]) OR (mitral valve regurgitation）**** Sort by: ****Most Recent**** | [42,162](https://pubmed.ncbi.nlm.nih.gov/?term=longqueryb056fd93afadb48a40ff&sort=date&size=200) | 08:13:59 |
| #1 |  |  | Search: ****"Mitral Valve Insufficiency"[Mesh]**** Sort by: ****Most Recent**** |  |  |
